# GCGFormer: Geometry-Compatibility-Guided Attention for Rotated Object Detection

**DOI:** 10.3390/s26195995

**Published:** 2026-09-22

**Authors:** Huiji Wang, Dandan Huang, Zhi Liu, Zhichao Han, Yunhe Qiu, Chunpeng Pan

**Affiliations:** 1 School of Electronic Information Engineering, Changchun University of Science and Technology, Changchun 130022, China; hjwang@mails.cust.edu.cn (H.W.); hdd@cust.edu.cn (D.H.); zchan@mails.cust.edu.cn (Z.H.); qiuyunhe@mails.cust.edu.cn (Y.Q.); 2024100988@mails.cust.edu.cn (C.P.); 2 Institute of Space Optoelectronics Technology, Changchun University of Science and Technology, Changchun 130022, China; 3 Jilin Provincial Urban Water Supply Co., Ltd., Changchun 130000, China

**Keywords:** remote sensing, rotated object detection, dual token representation, geometry compatibility, attention mechanism

## Abstract

Rotated object detection in remote sensing imagery requires both semantic understanding and accurate geometric reasoning. Existing geometry-aware detectors commonly introduce geometric information through oriented proposals or decoder queries, making their designs dependent on specific detection architectures, while geometric relationships among feature tokens remain insufficiently explored. To address this limitation, we propose GCGFormer, a geometry-compatibility-guided attention module for rotated object detection. Specifically, GCGFormer constructs a dual-token representation and a geometry compatibility matrix to model pairwise spatial relationships, including positional proximity, aspect ratio consistency, and orientation alignment. The matrix is integrated into self-attention to jointly optimize semantic similarity and geometric coherence. The proposed module can be readily integrated into existing detectors without extensive modifications. As an architecture-independent module placed before the detection head, GCGFormer can be integrated into different rotated object detectors. Experiments on DOTA-v1.0 show that GCGFormer improves the mAP of Oriented R-CNN from 65.1% to 65.6%, Rotated Faster R-CNN from 63.3% to 63.8%, Rotated ATSS from 61.7% to 62.1%, S2ANet from 62.7% to 63.1%, and YOLOv8-OBB from 72.7% to 72.9%. These consistent, although modest, improvements demonstrate that explicit feature-token geometric compatibility provides a generally applicable complement to semantic attention for rotated object detection.

## 1. Introduction

Object detection in remote sensing imagery is fundamental for applications such as urban monitoring, traffic analysis, environmental surveillance, and disaster assessment [1,2]. Compared with natural images, remote sensing imagery presents unique challenges, including large-scale variations, cluttered backgrounds, dense object distributions, and arbitrary object orientations [3,4]. These characteristics make rotated object detection particularly difficult, as accurate localization requires precise modeling of both semantic appearance and geometric structure.

To achieve tighter localization, rotated object detection methods typically employ oriented bounding boxes (OBBs), parameterized by object position, spatial extent, and rotation angle [5,6]. Nevertheless, they also introduce additional difficulties, including angular periodicity, boundary discontinuity, extreme aspect ratios, and feature misalignment. Consequently, effective rotated detection requires the joint modeling of semantic content and geometric structure.

Convolutional neural networks (CNNs) have been widely adopted for rotated object detection due to their hierarchical representation capability [7,8,9]. These approaches have established effective detection pipelines, but their geometric mechanisms are generally coupled with particular backbones, proposal representations, RoI operations, or detection heads. This coupling limits the direct reuse of the same geometric-relation formulation across heterogeneous detector architectures.

Recently, Transformer-based architectures have shown promising performance by leveraging self-attention mechanisms to capture global contextual interactions [10,11]. Recent studies have therefore introduced explicit geometry or orientation priors into Transformer-based rotated detectors. AO2-DETR employs oriented proposal generation, adaptive proposal refinement, and rotation-aware set matching [12]. OrientedFormer represents oriented-box queries using Gaussian distributions and introduces Gaussian positional encoding, Wasserstein self-attention, and oriented cross-attention [13]. GA-DETR further investigates geometry-aware mechanisms for aerial oriented detection [14], while RS-DETR and OPT-Net incorporate rotation–semantic interaction and orientation-preserving query processing into their respective decoder frameworks [15,16]. These studies demonstrate that explicit geometry can benefit rotated detection; therefore, the remaining issue is not whether geometry can be introduced into attention, but how geometric compatibility can be modeled at the feature-token level and reused across different detector paradigms.

Most existing geometry-aware Transformer detectors derive geometric information from object queries, oriented proposals, or intermediate decoder states. Their mechanisms are therefore closely associated with query initialization, decoder self- or cross-attention, iterative box refinement, or bipartite matching. In contrast, a detector-independent mechanism operating before the detection head should model geometric relationships directly among feature tokens without requiring a redesign of the detector’s native prediction process. Such a mechanism must also address two practical concerns. First, the learned geometric attributes should correspond to meaningful object properties rather than unconstrained latent variables. Second, geometric priors should guide rather than overwhelm the semantic attention logits.

To address these issues, we propose GCGFormer, a geometry-compatibility-guided attention module for rotated object detection. GCGFormer adopts separate semantic and geometric token representations. The geometric encoder uses a token-level two-layer multilayer perceptron to produce position, aspect-ratio, and orientation descriptors. The geometric constraints are constructed from geometric consistency and an auxiliary geometric loss. Based on these attributes, GCGFormer constructs a pairwise compatibility matrix that jointly describes positional proximity, aspect-ratio consistency, and orientation alignment. The matrix is incorporated into the semantic attention logits through a bounded learnable scalar Gate, allowing the contribution of geometric compatibility to be adjusted during training.

The proposed method is evaluated locally on the DOTA-v1.0 validation split. All improvements are calculated between each reproduced baseline and its GCGFormer-enhanced counterpart under identical training and evaluation settings. Across five detector architectures, the matched comparisons show modest improvements of 0.2–0.5 mAP. Complexity measurements show that GCGFormer adds 0.29 M parameters and 7.49 GFLOPs to the evaluated baseline.

The main contributions of this work are summarized as follows.

- We introduce a feature-token-level geometric compatibility bias for rotated object detection. Unlike detector-specific methods operating on oriented proposals or decoder queries, GCGFormer is applied before the detection head and can be incorporated into heterogeneous rotated detector architectures.
- We construct a unified compatibility matrix using positional proximity, aspect-ratio consistency, and periodic orientation alignment. A geometric encoder, geometric loss, normalized geometric weighting, and a bounded scalar Gate are employed to improve the interpretability of the geometric attributes and control their influence on semantic attention.
- We evaluate GCGFormer on the DOTA-v1.0 validation split using two-stage, one-stage anchor-based, and one-stage anchor-free detectors. The evaluation includes matched baseline comparisons, geometric-factor and Gate ablations, repeated runs with different random seeds, and quantitative measurements of parameters, GFLOPs, and inference speed, providing evidence of modest performance improvements and cross-architecture applicability.

## 2. Related Work

### 2.1. Self-Attention in Vision Transformers

Self-attention, originally proposed in Transformer [17] for natural language processing, enables long-range dependency modeling and overcomes the local receptive field limitation of CNNs. Vision Transformer (ViT) [11] first introduced vanilla self-attention into visual feature learning, but its quadratic complexity and limited spatial structural awareness restrict high-resolution applications. To improve efficiency, Swin Transformer [18], Linear Transformer [19], and FlashAttention [20] introduced sparse or linearized attention mechanisms. Other methods, such as CBAM [21], Coordinate Attention [22], and MobileViT [23], enhanced spatial representation by integrating local feature modeling. However, most existing approaches mainly focus on semantic similarity and lack explicit geometric compatibility modeling.

### 2.2. Geometry-Aware Vision Models

Geometry-aware modeling has been widely explored in computer vision, yet most methods incorporate geometric information through auxiliary modules rather than directly embedding it into Transformer attention mechanisms. Geoformer [24] introduced geometry-guided attention for 3D point cloud detection, while GeoViT [25] integrated depth and surface normal priors into Vision Transformers for scene understanding. LatFormer [26] improved geometric generalization by embedding lattice symmetry priors into attention masks, and G2VLM [27] achieved spatial reasoning through geometry-semantic cross-modal learning. However, these methods mainly focus on natural scenes or 3D tasks and lack targeted modeling for arbitrarily oriented and densely distributed objects in remote sensing imagery. BEI-Net [28] for RGB-T camouflaged object detection employed edge-aware supervision and cross-band interaction to alleviate boundary inconsistencies and suppress misleading edge cues; this work demonstrates the value of structural priors for feature interaction. The existing GeoFormer is designed for point-cloud-based 3D object detection and uses a graph-based Geometry Point Encoder to model geometric relations among points. In contrast, GCGFormer operates on two-dimensional feature tokens before the detection head and introduces a pairwise geometric compatibility matrix as an attention-logit bias for rotated object detection. Moreover, GCGFormer is designed as a detector-independent module that can be integrated into two-stage, one-stage, and anchor-free detectors.

### 2.3. Remote Sensing Rotated Object Detection

Remote sensing rotated object detection aims to address arbitrary orientations, dense object distributions, and large aspect-ratio variations in aerial imagery. Early methods such as RRPN [7] and R3Det [6] established anchor-based rotated detection frameworks through rotated proposals and coarse-to-fine regression strategies. To enhance geometric representation, CNN-based approaches including ReDet [29] and LSKNet [30] introduced rotation-equivariant modeling and adaptive receptive field mechanisms for improved spatial awareness and contextual representation. Recently, anchor-free detectors such as PP-YOLOE-R [31] achieved efficient and deployment-friendly rotated detection through angle-aware regression and alignment strategies. Transformer-based methods, represented by O^2^-DETR [32], further improved global contextual modeling for oriented detection. However, most existing approaches introduce geometry through auxiliary encoding or optimization strategies rather than directly embedding geometric priors into the attention mechanism. In contrast, GCGFormer explicitly incorporates geometric consistency into attention computation, enabling more effective geometry-aware reasoning for rotated object detection.

## 3. Method

### 3.1. Overall Framework

To effectively model arbitrarily oriented objects in remote sensing imagery, we propose GCGFormer, a geometry-aware Transformer framework that explicitly incorporates geometric reasoning into feature interaction. As illustrated in Figure 1, GCGFormer consists of four key components: Dual Token Construction, Geo-compatibility Matrix, Geometric Consistency Constraints and Geometry-aware Attention.

Given an input feature map ($X\in R^{H\times W\times C}$), GCGFormer adopts a dual-branch architecture to construct semantic and geometric token representations. The semantic branch generates Query, Key, and Value embeddings for contextual interaction, while the geometric branch extracts spatial attributes, including position, aspect ratio, and orientation. Based on these attributes, a geometry compatibility matrix ($G\in R^{N\times N}$) is constructed to model pairwise geometric relationships among tokens. The matrix is further integrated into the attention computation to guide feature aggregation with geometric consistency constraints. Finally, the geometry-enhanced features are refined through FFN and residual connections, producing more discriminative and geometry-aware representations for rotated object detection.

### 3.2. Dual Token Construction

The proposed design explicitly decouples semantic and geometric representations into two parallel branches, thereby enabling the network to simultaneously capture contextual semantics and spatial relational cues. This dual-token-branch formulation provides a more comprehensive feature representation for arbitrarily oriented objects in remote sensing imagery.

Given an input feature map ($X\in R^{H\times W\times C}$), where (N=H∗W) denotes the number of tokens and (*C*) represents the feature dimension, GCGFormer first projects the input features into two complementary token spaces: the semantic token space and the geometric token space.

a. Semantic Token Branch

In the semantic branch, the input feature ($X\in R^{H\times W\times C}$) is projected into semantic tokens ($T_{s}$) through learnable linear transformations, which are further used to generate the Q, K and V embeddings for feature interaction. This branch mainly captures global contextual dependencies and appearance similarity among objects through semantic feature aggregation.

b. Geometry Token Branch

Meanwhile, the geometric branch is introduced to explicitly encode the structural properties of rotated objects. Specifically, a Geometric Encoder extracts geometric attributes from each token, including spatial position, object aspect ratio, and rotation orientation. The resulting geometric token can be expressed as

(1) $$T_{g}=\phi_{g}\left(X\right)$$

where $\phi_{g}\left(\cdot \right)$ denotes Geometry Encoder.

c. Geometry Encoder

In Figure 2, given the input feature tokens $\left(X_{t}\in R^{B\times N\times C}\right)$, the geometric encoder $\left(\phi_{g}\right)$ consists of two linear layers and a ReLU activation:

(2) $${\mathrm{Linear}}_{128\to 5}\left[\mathrm{ReLU}\left({\mathrm{Linear}}_{C\to 128}\left(X_{t}\right)\right)\right]$$

where (N) is the number of feature tokens, and (C) is the input channel dimension. The intermediate representation has a dimension of (N×128), and the resulting geometric token representation is $\left(T_{g}\in R^{N\times 5}\right)$. The position, orientation and aspect-ratio attributes used to construct the geometric compatibility matrix are decoded from $\left(T_{g}\right)$.

(3) $$\left(x_{i},y_{i},r_{i},cos2\theta_{i},sin2\theta_{i}\right)=\phi_{g}\left(X_{i}\right)$$

where x,y are the coordinates of the object’s center, r is the aspect ratio, and θ is the rotation angle.

It should be noted that the geometric attributes are not obtained from detection heads, anchors, or proposal boxes. Instead, they are estimated directly from backbone features through the proposed geometric encoder and are optimized jointly with the detection objective in an end-to-end manner.

The proposed dual-token construction enables GCGFormer to jointly model semantic context and geometric structure within a unified framework. By decoupling appearance representation from spatial reasoning, the network can more effectively capture orientation-sensitive and geometry-consistent relationships among arbitrarily oriented objects. This design further provides the foundation for subsequent geometry-aware attention computation.

### 3.3. Auxiliary Geometric Supervision

Optimizing the geometric encoder solely through the detection objective does not guarantee that its predicted orientation attribute corresponds to a physically meaningful rotation angle. Therefore, we introduce an auxiliary geometric loss to directly constrain the orientation and aspect-ratio attributes predicted by the geometric encoder.

For each positive RoI, the corresponding ground-truth oriented bounding box is assigned as the geometric supervision signal for the feature tokens inside that RoI.

The orientation is represented using the double-angle encoding:

(4) $$o_{i}=\frac{{\tilde{o}}_{i}}{\max\left(∥{\tilde{o}}_{i}{∥}_{2},\epsilon \right)}=\left[\cos\left(2{\hat{\theta }}_{i}\right),\sin\left(2{\hat{\theta }}_{i}\right)\right],\;o^{*}=\left[\cos\left(2\theta^{*}\right),\sin\left(2\theta^{*}\right)\right],$$

where $\left({\tilde{o}}_{i}\right)$ is the raw orientation vector predicted by the Geometry Encoder, $\left(o_{i}\right)$ is the predicted orientation vector of the (i)-th token and (o) is obtained from the ground-truth rotation angle of the corresponding RoI. The double-angle representation respects the (π)-periodicity of oriented bounding boxes and avoids discontinuity at the angular boundary.

The predicted and ground-truth aspect ratios are denoted as:

(5) $$r^{*}=\mathrm{Sigmoid}\left(\log\frac{w^{*}}{h^{*}}\right)=\frac{w^{*}}{w^{*}+h^{*}}$$

where ${\tilde{r}}_{i}$ is the unconstrained aspect-ratio logit predicted by the Geometry Encoder, and $r_{i}=\mathrm{Sigmoid}\left({\tilde{r}}_{i}\right)\in \left(0,1\right)$ is its normalized value.

Because tokens near the RoI boundary may contain more background or neighboring-instance information, a Gaussian spatial weight $\left(s_{i}\right)$ is introduced to assign stronger supervision to tokens near the RoI center. The auxiliary geometric loss is defined as:

(6) $$L_{\mathrm{geo}}=\frac{\sum_{i=1}^{N}s_{i}\left[\lambda_{\theta }\left(1-oi^{⊤}o^{*}\right)+\lambda_{r}\mathrm{SmoothL1}\left(r_{i},r^{*}\right)\right]}{\sum_{i=1}^{N}s_{i}+\epsilon }$$

where (N) is the number of tokens inside the RoI, $\left(\lambda_{\theta }\right)$ and $\left(\lambda_{r}\right)$ balance the orientation and aspect-ratio terms, and (ϵ) ensures numerical stability. This loss is calculated only for positive RoIs. The complete training objective is:

(7) $$L_{\mathrm{total}}=L_{\det}+\lambda_{\mathrm{aux}}L_{\mathrm{geo}}$$

where $\left(L_{\det}\right)$ denotes the original detector loss and $\left(\lambda_{\mathrm{aux}}\right)$ controls the contribution of geometric supervision. This supervision improves the interpretability of the predicted attributes but does not constitute an independent quantitative evaluation of their reliability.

### 3.4. Geometric Consistency Constraints

To enhance geometry-aware feature interaction for rotated object detection, GCGFormer introduces Geometric Consistency Constraints (GCC) to explicitly model the spatial structural relationships among tokens. Specifically, GCC is composed of three complementary geometric components: Position Proximity, which characterizes spatial adjacency between objects; Aspect Ratio Consistency, which models structural shape similarity; and Orientation Alignment, which captures directional coherence among rotated targets. By jointly considering these geometric constraints, GCGFormer provides effective spatial priors for subsequent geometry-aware relational reasoning and feature aggregation.

#### 3.4.1. Position Proximity

Given the center coordinates of two tokens $p_{i}=\left(x_{i},y_{i}\right)$ and $p_{j}=\left(x_{j},y_{j}\right)$, the positional compatibility is formulated as

(8) $$G_{p}\left(i,j\right)=\exp\left(-\frac{∥p_{i}-p_{j}{∥}^{2}}{\sigma_{p}^{2}}\right)$$

where $\sigma_{p}$ is a scaling factor controlling the sensitivity to spatial distance. This formulation assigns higher compatibility scores to spatially closer tokens while suppressing interactions between distant regions.

The position consistency constraint effectively enhances local structural coherence and reduces irrelevant long-range associations. However, spatial proximity alone is insufficient for accurately characterizing rotated objects, particularly in densely distributed scenes where multiple objects may be spatially close but geometrically unrelated.

#### 3.4.2. Aspect Ratio Consistency

In addition to spatial position, object shape serves as a crucial structural prior. Thus, we adopt aspect ratio consistency constraint in GCGFormer to model token shape similarity.

Let $r_{i}$ and $r_{j}$ denote the aspect ratio descriptors of two tokens, where the aspect ratio is defined as the ratio between object width and height. The compatibility between two tokens is formulated as

(9) $$G_{ar}\left(i,j\right)=\exp\left(-\frac{|r_{i}-r_{j}|}{\sigma_{r}}\right)$$

where $\sigma_{r}$ is a scaling parameter controlling the sensitivity to aspect ratio differences. Under this formulation, tokens with similar geometric proportions receive higher compatibility scores, while those with different shape structures are suppressed.

Aspect ratio consistency provides a more robust structural descriptor for rotated object detection than absolute aspect ratio information, as it is less sensitive to scale and resolution variations. It enables GCGFormer to capture structural similarity among objects with similar geometric proportions, improving discrimination between geometrically different targets. Combined with positional proximity and orientation alignment, it further provides effective geometric priors for geometry-aware relation modeling and attention computation.

#### 3.4.3. Orientation Alignment

Among all geometric factors, orientation consistency plays the most critical role in rotated object detection. Since remote sensing objects frequently appear at arbitrary orientations, directional alignment becomes a key indicator for determining whether two regions belong to the same structural pattern. To explicitly model directional relationships, GCGFormer introduces an orientation consistency constraint based on angular similarity. Let $\theta_{i}$ and $\theta_{j}$ denote the rotation angles of two tokens. The orientation compatibility is computed as

(10) $$G_{o}\left(i,j\right)=\frac{1+o_{i}^{T}o_{j}}{2}=\left(1+\cos2\left(\theta_{i}-\theta_{j}\right)\right)/2$$

where $o_{i}=\left[\cos\left(2\theta_{i}\right),\sin\left(2\theta_{i}\right)\right]$. This formulation maps the orientation similarity to [0, 1]. Since o(θ+π)=o(θ), it is invariant to a ($180^{\circ }$) rotation and avoids discontinuities at angular boundaries. The GCM directly uses the orientation-vector inner product; therefore, the scalar angles do not need to be recovered from the double-angle representation.

The orientation constraint improves the network’s ability to model elongated and direction-sensitive structures, such as bridges, ships, and runways. Moreover, it effectively reduces background interference caused by semantically similar but directionally inconsistent regions. Experimental results in the ablation study further demonstrate that orientation consistency contributes the largest performance gain among all geometric factors, highlighting its importance in geometry-aware attention modeling.

### 3.5. Geometric Compatibility Matrix

The Geo-compatibility Matrix ($G\in R^{N\times N}$) is introduced as a geometry-guided relational prior to regulate token interactions in rotated object detection. Each element $G_{ij}$ measures the geometric compatibility between token *i* and token *j*, encouraging interactions among geometrically coherent regions while suppressing irrelevant associations. This explicit modeling of geometric structure enhances the capability to distinguish densely distributed and arbitrarily oriented objects in remote sensing imagery.

Unlike conventional positional encoding, which embeds geometric information implicitly into feature representations, the proposed formulation adopts an explicit pairwise strategy to directly model geometric dependencies, thereby improving robustness in structured spatial reasoning tasks.

All three compatibility terms are now bounded within [0,1]. Their contributions are further controlled by softmax-normalized weights:

(11) $$\left[w_{p},w_{o},w_{r}\right]=Softmax\left(\left[a_{p},a_{o},a_{r}\right]\right)$$

where $\left(w_{p},w_{o},w_{r}\geq 0\right)$ and $\left(w_{p}+w_{o}+w_{r}=1\right)$.

Given two tokens *i* and *j*, the geometric compatibility score is defined as

(12) $$G_{ij}=w_{p}G_{p}\left(i,j\right)+w_{o}G_{o}\left(i,j\right)+w_{r}G_{ar}\left(i,j\right)$$

where $G_{p}\left(i,j\right)$, $G_{o}\left(i,j\right)$, and $G_{ar}\left(i,j\right)$ denote position, orientation, and aspect ratio compatibility, respectively. The coefficients $w_{p}$, $w_{o}$, and $w_{r}$ are learnable parameters that adaptively balance different geometric factors during optimization. The geometric weights are initialized equally and optimized jointly with the detector using backpropagation.

Specifically, the position term captures spatial locality by measuring the distance between token centers, promoting interactions among nearby regions. The orientation term models directional consistency, reducing associations between geometrically misaligned objects. The aspect ratio term introduces shape awareness, enabling discrimination between objects with similar semantics but different geometric structures.

From a theoretical perspective, the Geo-compatibility Matrix can be interpreted as a geometry-guided structural prior imposed on the attention space. In standard self-attention, interactions are determined solely by semantic similarity, which may lead to ambiguous feature aggregation in cluttered scenes. By incorporating geometric constraints, the proposed method biases attention toward geometrically coherent regions, improving the reliability of feature interaction.

Furthermore, the adaptive weighting mechanism allows the model to learn the relative importance of different geometric factors. In practice, orientation consistency contributes to rotated object detection, while position and aspect ratio serve as complementary constraints.

The matrix-based formulation is computationally efficient and introduces modest overhead, making it suitable for integration into various Transformer-based detection frameworks. Overall, the Geo-compatibility Matrix enables explicit geometric relational modeling, effectively bridging semantic representation and spatial structure reasoning for improved rotated object detection performance.

### 3.6. Geometry-Aware Attention

Based on the constructed Geo-compatibility Matrix, GCGFormer introduces a Geometry-aware Attention (GA) mechanism to incorporate geometric constraints into the self-attention process. The objective of this module is to jointly model semantic similarity and geometric consistency, thereby improving feature aggregation for arbitrarily oriented objects in complex remote sensing scenes.

In conventional self-attention, feature interactions are computed solely according to semantic similarity between tokens. Given Query (Q), Key (K), and Value (V), the standard attention is formulated as

(13) $$A_{s}=\frac{QK^{⊤}}{\sqrt{d}}$$

where *d* denotes the channel dimension. Although effective for capturing long-range dependencies, this formulation lacks explicit geometric reasoning capability. Consequently, semantically similar but geometrically inconsistent regions may still produce attention responses, resulting in ambiguous feature aggregation in cluttered remote sensing imagery.

To address this limitation, the proposed GCGFormer introduces the Geo-compatibility Matrix (G) to modulate the semantic attention distribution; the normalized geometry-aware attention is then computed as:

(14) $$A_{g}=\mathrm{Softmax}\left(A_{s}⊕\left(\mathrm{Gate}\cdot G\right)\right)$$

where ⊕ denotes element-wise addition.

The Gate is parameterized by a single unconstrained scalar $\left(\gamma \in R^{1}\right)$, and its effective value is obtained using the sigmoid function: g=sigmoid(γ),g∈(0,1). The scalar (g) is shared across all attention heads and token pairs within the GCGFormer module and is broadcast over the geometric compatibility matrix $\left(G\in R^{N\times N}\right)$. The raw parameter (γ) is initialized to the constant value (0), yielding an initial Gate value of (g=0.5), and is optimized jointly with the detector during training.

Through this operation, semantic attention scores are dynamically adjusted according to the geometric compatibility between token pairs. The Gate controls the contribution of geometric compatibility during attention computation and prevents geometry constraints from overwhelming semantic similarity. The final feature aggregation is formulated as:

(15) $$Y=A_{g}V\;$$

where *Y* denotes the geometry-enhanced feature representation. The output features are further refined using feed-forward networks and residual connections to stabilize optimization and preserve contextual information.

Compared with standard self-attention, the proposed Geometry-aware Attention explicitly introduces geometric relational constraints into the attention computation, enabling structured spatial reasoning beyond appearance similarity. By emphasizing geometrically coherent regions and suppressing inconsistent feature interactions, the proposed mechanism improves orientation sensitivity and localization robustness for rotated objects.

Moreover, the matrix-based formulation introduces modest computational overhead, allowing GCGFormer to be seamlessly integrated into various Transformer-based detection frameworks. In general, the proposed mechanism effectively bridges semantic representation learning and geometric structure modeling for rotated object detection in remote sensing imagery. The pseudo-code of the proposed GCGFormer Geometry-aware Attention module is presented in Algorithm A1.

### 3.7. Integration of GCGFormer into Different Detection Frameworks

GCGFormer is designed as a plug-and-play module that can be seamlessly integrated into various rotated object detection frameworks without substantial architectural modifications. Using GCGFormer, it enhances spatial structural representation and improves localization performance for arbitrarily oriented objects. To ensure architectural consistency and deployment flexibility, GCGFormer is inserted uniformly before the detection heads of different detectors (two-stage detectors and one-stage detectors). GCGFormer is integrated before dense prediction heads. Experimental results demonstrate consistent performance gains across different detector paradigms, validating the adaptability and cross-architecture applicability of the proposed geometry-aware attention mechanism.

## 4. Experiments

To comprehensively validate the effectiveness, cross-architecture applicability, and rationality of the proposed GCGFormer module for rotated object detection in remote sensing images, a series of experiments are conducted on the DOTA benchmark dataset. This section elaborates on the experimental settings, ablation studies, comparative results embedded within different detection frameworks, visual analysis, and in-depth discussion of the proposed method. All experiments follow a unified training and testing pipeline to guarantee the fairness and reliability of the experimental evaluation.

### 4.1. Datasets and Implementation Details

#### 4.1.1. Dataset

The experiments are conducted on the DOTA-v1.0 benchmark, one of the most widely used and representative datasets for rotated object detection in remote sensing imagery. The dataset contains annotated objects belonging to 15 categories, including typical remote sensing targets such as airplanes, ships, bridges, harbors, vehicles, and baseball diamonds. Most of these objects exhibit arbitrary orientations and aspect ratio variations, which are consistent with the practical challenges encountered in rotated object detection tasks:

Plane (PL), Baseball diamond (BD), Bridge (BR), Ground track field (GTF), Small vehicle (SV), Large vehicle (LV), Ship (SH), Tennis court (TC), Basketball court (BC), Storage tank (ST), Soccer-ball field (SBF), Roundabout (RA), Harbor (HA), Swimming pool (SP), and Helicopter (HC).

#### 4.1.2. Implementation Details

All experiments are implemented with the PyTorch deep learning framework. The hardware and software configurations are summarized as follows: GPU: NVIDIA RTX 4090D; CUDA version: 11.3; PyTorch version: 1.10; Python version: 3.8. The input image size is set to 1024 × 1024 pixels. We employ both SGD and AdamW as optimizers for model training. The parameters involved in the experiment are: $\lambda_{\theta }=1$, $\lambda_{r}=1$, $\lambda_{aux}=0.2$, $\sigma_{p}=1$, $\sigma_{r}=1$, ϵ = 1 $\times \;10^{-8}$.

The key training hyperparameters are set consistently for all models to ensure fair comparisons: the batch size is set to 8, the initial learning rate is 0.001 (optimized using the SGD optimizer with momentum = 0.9 and weight decay = 0.0001, optimized using the AdamW optimizer with momentum = 0.9 and weight decay = 0.0005), and the total training epochs are 12. A learning rate decay strategy is adopted, where the learning rate is reduced by a factor of 10 at the 8th and 10th epochs. No additional post-processing techniques are used during testing to avoid introducing extra factors that may affect the evaluation results.

### 4.2. Ablation Study

In this section, we conduct comprehensive ablation studies on the DOTA dataset to systematically evaluate the effectiveness of the proposed GCGFormer module. The experiments are built upon the Oriented R-CNN baseline, and all training settings are kept identical to ensure fair comparisons. By progressively enabling or disabling different components of GCGFormer, we isolate the contributions of individual geometric factors, analyze their interactions, and examine the overall design rationality of the proposed framework.

#### 4.2.1. Effect Study of GCGFormer Components

To evaluate the effectiveness of each core component in the proposed GCGFormer, we conduct a series of ablation experiments by progressively introducing Self-Attention (SA) and Geometry-aware Attention (GCGFormer) into the baseline model. The results are summarized in Table 1.

From the results, incorporating the standard self-attention (SA) module yields only a marginal improvement, increasing the mAP from 65.1% to 65.2% (+0.1%). Although self-attention enhances global contextual modeling by capturing long-range dependencies, its effectiveness remains limited without explicit geometric reasoning. In rotated object detection, where targets exhibit arbitrary orientations and complex spatial distributions, semantic similarity alone is insufficient for reliable feature aggregation.

In contrast, introducing the complete GCGFormer module improves the performance to 65.6% mAP, achieving a gain of +0.5% over the baseline. This improvement validates the effectiveness of the proposed geometry-aware attention mechanism. By explicitly modeling geometric relationships and integrating them into the attention computation, GCGFormer enables the network to jointly consider semantic consistency and spatial structural coherence. Consequently, the model can better distinguish geometrically relevant regions from misleading background features.

Furthermore, the clear performance gap between the “+SA” and “+GCGFormer” settings indicates that the improvement is not simply attributed to the use of attention mechanisms, but primarily arises from the incorporation of geometry-aware constraints into the attention process. This observation further confirms the importance of geometric reasoning in rotated object detection and supports the design motivation of GCGFormer.

In conclusion, the results demonstrate that while standard self-attention provides limited gains in contextual modeling, the proposed GCGFormer improves detection performance through explicit geometric relational modeling.

As shown in Figure 3a, the prominent diagonal of *G* represents the high self-compatibility of individual tokens, while the off-diagonal banded and block-wise patterns indicate groups of tokens with similar positional, orientation, and aspect-ratio attributes. Compared with the baseline attention map in Figure 3b, the GCGFormer map in Figure 3c exhibits more spatially coherent responses along oriented structures and object boundaries. This qualitative comparison suggests that the geometric compatibility matrix reorganizes token interactions by strengthening geometrically consistent relationships. Nevertheless, the visualization is interpretive and should be considered together with the quantitative ablation results.

#### 4.2.2. Effect of Individual Geometric Factors

We first investigate the individual contributions of three fundamental geometric factors in GCGFormer, namely position, aspect ratio, and orientation. To this end, we design a set of single-factor ablation experiments, where each factor is independently incorporated into the model. The quantitative results are summarized in Table 2.

The results reveal that different geometric factors contribute unequally to the overall detection performance. Among them, the orientation factor yields the most improvement, boosting the mAP from 65.1% to 65.5% (+0.4%). This observation highlights the critical role of orientation consistency in rotated object detection, where objects often exhibit arbitrary rotations. The lack of explicit orientation modeling in standard attention mechanisms tends to introduce incorrect associations, especially in densely distributed or cluttered scenes. By explicitly encoding angular relationships, GCGFormer effectively mitigates such issues and enhances localization accuracy.

In contrast, the position factor provides only a marginal improvement (+0.1%), indicating that spatial proximity alone offers limited discriminative power for feature aggregation. Similarly, the aspect ratio factor does not bring noticeable gains when used independently, suggesting that aspect ratio information alone is insufficient to guide reliable attention learning. These findings imply that while position and aspect ratio contain useful geometric cues, their effectiveness largely depends on interactions with other factors.

Aspect-ratio compatibility alone produces the same mAP as the baseline and therefore does not demonstrate an independent performance benefit. However, the third and fifth rows of Table 3 can be used as a comparison of the leave-one-out method experiments. The leave-one-out comparison shows that adding aspect-ratio compatibility to the position-and-orientation configuration increases mAP from 65.1% to 65.6%. This observation indicates that aspect ratio consistency mainly functions as a structural refinement term within the overall geometry-aware attention framework. In summary, this set of experiments demonstrates that orientation serves as the dominant geometric prior, while position plays a supplementary role and aspect ratio requires joint modeling to be effective.

#### 4.2.3. Effect of Geometric Factor Combinations

To further explore the interactions among different geometric factors, we conduct ablation experiments on their pairwise and full combinations. The results are reported in Table 3.

**Table 3:** Ablation study on their pairwise and full combinations. (The baseline is Oriented R-CNN on the DOTA dataset (using Seed 4)).

Model	Position	Aspect Ratio	Orientation	mAP (%)	Gain
Baseline				65.1	–
Baseline	✓	✓		65.2	+0.1
Baseline	✓		✓	65.3	+0.2
Baseline		✓	✓	65.2	+0.1
Baseline	✓	✓	✓	**65.6**	**+0.5**

The bold values represent the best performance.

Among the pairwise combinations, position plus orientation achieves the best performance of 65.3% mAP (+0.2), while position plus aspect ratio and aspect ratio plus orientation both achieve 65.2% (+0.1). This suggests that position and orientation provide relatively stronger complementary information, although the gains from all pairwise combinations remain modest.

In contrast, when all three factors are jointly considered, the model achieves the best performance of 65.6% mAP, corresponding to a +0.5% improvement over the baseline. This result clearly demonstrates that the effectiveness of geometric modeling lies in the joint representation of multiple factors rather than isolated contributions.

These findings validate that the proposed geometry compatibility modeling in GCGFormer is capable of capturing multi-dimensional geometric dependencies, enabling better feature aggregation for rotated object detection.

The ablation studies validate the effectiveness of GCGFormer from three perspectives: module design, geometric factor contribution, and multi-factor interaction. Standard self-attention provides only limited improvement, whereas GCGFormer enhances detection performance through geometry-aware feature aggregation. Furthermore, the best performance is achieved by jointly combining position, orientation, and aspect ratio constraints, demonstrating that multi-dimensional geometric cues are complementary and essential for spatial structural reasoning.

#### 4.2.4. Effect of Gate Mechanism Combinations

To ensure transparent comparison, the random seed used for each configuration is reported explicitly in Table 4. The geometric-weight ablation was conducted using Seed 4. Under this setting, fixed equal weights achieve 65.3% mAP, whereas normalized learnable weights achieve 65.60% mAP. The fixed-Gate configurations were evaluated using Seed 4 and are identified separately in the table.

As shown in Table 4, the learnable Gate achieves the highest mAP of 65.6, exceeding both fixed settings (g=0 and g=1) by 0.4 mAP points. The identical performance of the two fixed settings indicates that neither completely suppressing geometric compatibility nor incorporating it at full strength provides an optimal balance. By adaptively controlling the contribution of geometric compatibility, the learnable Gate better coordinates geometric constraints with semantic similarity, thereby improving detection performance.

### 4.3. Main Results

Unless otherwise specified, all reproduced baseline and GCGFormer-enhanced results reported in Table 4 and Table 5 were evaluated locally on the DOTA-v1.0 validation split and were not submitted to the official DOTA test server. Accordingly, the effectiveness of GCGFormer is assessed exclusively by comparing each in-house baseline with its corresponding GCGFormer-enhanced model under identical experimental settings. Published test-set results are included solely as external references and are neither directly compared with our validation-set results nor used to calculate the reported improvements.

#### 4.3.1. Cross-Architecture Applicability on Detectors

To evaluate the cross-architecture applicability of the proposed GCGFormer, we further integrate the module into multiple representative rotated object detectors, including two-stage detectors (Rotated Faster R-CNN and Oriented R-CNN) and one-stage detectors (Rotated ATSS, YOLOv8-OBB and S2ANet). Figure 4 illustrates the deployment of the GCGFormer module on different algorithms. All experiments are conducted on the DOTA dataset under identical training settings to ensure fair comparison. The quantitative results are summarized in Table 5.

The experimental results demonstrate that GCGFormer consistently improves the detection performance across all detector architectures. Specifically, Rotated Faster R-CNN achieves an improvement from 63.3% to 63.8% mAP, yielding a gain of +0.5%. Oriented R-CNN improves from 65.1% to 65.6%, while Rotated ATSS and S2ANet both obtain +0.4% mAP improvements. Even for the one-stage baseline YOLOv8-OBB, GCGFormer still achieves a noticeable improvement of +0.2% mAP.

These results indicate that the proposed geometry-aware attention mechanism exhibits architectural compatibility and is not restricted to a specific detection paradigm. The consistent gains across heterogeneous frameworks confirm that the effectiveness of GCGFormer originates from its explicit geometric relational modeling rather than detector-specific design choices. By introducing geometry consistency constraints into the attention computation, GCGFormer enhances feature aggregation in a unified manner for proposal-based, anchor-free, and dense prediction frameworks.

In addition, the improvements achieved on different detectors demonstrate that geometric reasoning serves as a universal and complementary cue for rotated object detection. In particular, larger gains observed in two-stage detectors suggest that proposal-based frameworks benefit more from geometry-enhanced feature representation during region localization and proposal refinement. Meanwhile, the improvements on one-stage and anchor-free detectors further verify the robustness and flexibility of the proposed module.

In summary, the above experiments validate the cross-architecture applicability and plug-and-play property of GCGFormer. Without introducing substantial architectural modifications or computational overhead, the proposed module can be seamlessly integrated into diverse detection frameworks and consistently improve rotated object detection performance in complex remote sensing scenarios.

#### 4.3.2. Quantitative Comparison Across Different Detectors

Table 6 presents the detection results of the 15 object categories in the DOTA dataset, demonstrating the effectiveness of GCGFormer across different detector architectures and object types.

Table 6 presents the class-wise results of the reproduced detectors before and after integrating GCGFormer. Although GCGFormer improves the overall mAP of all evaluated baselines, the class-level changes are not uniformly positive. The results therefore suggest that the effectiveness of geometric compatibility modeling depends on both the detector architecture and the geometric characteristics of individual categories.

From the category-wise results, GCGFormer exhibits particularly notable improvements on classes with geometric characteristics, such as HA, HC and BC. For example, under Rotated Faster R-CNN, the performance on BC improves from 54.7% to 61.7%, while HC increases from 54.0% to 56.6%. Similar trends can also be observed in Oriented R-CNN and YOLOv8-OBB. By explicitly incorporating geometric compatibility into the attention computation, GCGFormer enables the detector to better capture structural dependencies among rotated objects, thereby improving both classification and bounding box regression accuracy.

It is also worth noting that the proposed module introduces modest computational overhead while consistently improving performance across different detection paradigms. This demonstrates that the effectiveness of GCGFormer originates from its geometry-aware relational reasoning mechanism rather than detector-specific optimization strategies.

However, for Rotated Faster R-CNN, the overall mAP increases from 63.3% to 63.8%; however, the AP for ships (SH) decreases substantially from 78.4% to 71.2%, corresponding to a decline of 7.2 percentage points. This result is noteworthy because ships are typically elongated and orientation-sensitive objects for which explicit geometric modeling might be expected to be beneficial. A possible explanation is that ships in DOTA images frequently appear as densely arranged neighboring instances with similar shapes and orientations. Under such conditions, high geometric compatibility may be assigned not only to tokens belonging to the same ship but also to tokens from adjacent ships. The resulting cross-instance feature aggregation may weaken instance boundaries and adversely affect localization or classification. In addition, inaccurate token-level orientation estimates and the limited spatial resolution of RoI-aligned features may further reduce the reliability of the geometric prior. These explanations represent possible mechanisms rather than experimentally confirmed causal conclusions.

A similarly non-uniform pattern is observed for YOLOv8-OBB. Although its overall mAP increases from 72.7% to 72.9%, nine of the fifteen categories exhibit decreased AP values. Meanwhile, some categories show relatively large positive changes, including an increase of 7.0 percentage points for basketball courts (BC). The simultaneous occurrence of category-level gains and declines indicates that the modest overall improvement is produced by uneven class-wise changes rather than consistent improvements across all object categories. Categories with regular spatial layouts or stable geometric structures may benefit more from the compatibility prior, whereas densely distributed objects, objects with ambiguous orientations, and categories with substantial intra-class shape variation may be more sensitive to inaccurate geometric relationships.

All in all, the experimental results validate the effectiveness and plug-and-play capability of GCGFormer. The consistent improvements across multiple detectors and object categories confirm that explicitly modeling geometric consistency is highly beneficial for rotated object detection in complex remote sensing scenarios.

Consequently, the present experiments support the cross-architecture applicability of GCGFormer at the overall mAP level, but they do not demonstrate uniform class-wise improvements.

Table 7 presents representative published results of ReDet, O^2^-DETR, PP-YOLOE-R, and LSKNet on the DOTA-v1.0 test set. These methods report mAP values ranging from 79.66% to 81.85%, which are higher than those of our locally reproduced baselines. However, the published results were obtained using the official test server and different backbones, training schedules, data augmentation strategies, and testing settings. In contrast, our experiments were evaluated locally on the DOTA-v1.0 validation split. Therefore, these results are included only to clarify the absolute performance range of recent methods and are not used to calculate direct performance differences.

### 4.4. Computational Complexity Analysis

Table 1 reports the parameter count, computational cost, and inference speed of the baseline, standard self-attention (SA), and GCGFormer. Compared with the baseline, GCGFormer increases the number of parameters from 41.95 M to 42.24 M, corresponding to only 0.29 M additional parameters, or a relative increase of 0.69%. The computational cost increases from 199.58 to 207.07 GFLOPs, representing an additional 7.49 GFLOPs or 3.75%. Meanwhile, the detection accuracy increases from 65.1% to 65.6% mAP, yielding a gain of 0.5 percentage points. These results indicate that GCGFormer introduces limited parameter and arithmetic overhead while improving detection accuracy.

Compared with standard SA, GCGFormer achieves an additional improvement of 0.4 percentage points in mAP with only 0.03 M additional parameters. Its computational cost is 1.02 GFLOPs lower than that of SA, indicating that the accuracy improvement does not arise simply from using a more computationally expensive attention operation. However, the inference speed decreases from 32.13 FPS for the baseline and 29.86 FPS for SA to 26.64 FPS for GCGFormer. This corresponds to a 17.09% reduction relative to the baseline and a 10.78% reduction relative to SA. Overall, GCGFormer is parameter-efficient and introduces a modest increase in arithmetic complexity, but its runtime overhead is non-negligible.

### 4.5. Statistical Robustness Evaluation

To evaluate whether the improvement introduced by GCGFormer is robust to training variability, we repeated the baseline and GCGFormer-enhanced models using four matched random seeds (42, 2006, 13 and 4). Table 8 reports the results of the baseline and its GCGFormer-enhanced counterpart over four random seeds. The reproduced baseline achieves (64.99±0.20) mAP, whereas the model equipped with GCGFormer achieves (65.38±0.21) mAP. The corresponding paired improvement is (0.39±0.33) mAP.

GCGFormer produces positive gains under all four random seeds, ranging from (+0.10) to (+0.80) mAP. Moreover, the standard deviations of the baseline and GCGFormer-enhanced models are comparable, indicating that the introduction of the module does not noticeably increase the overall variability of the final detection accuracy. However, the magnitude of the improvement varies across seeds: the gains under Seeds 42 and 13 are relatively small, whereas Seeds 2006 and 4 yield larger improvements of (0.80) and (0.51) mAP, respectively.

Therefore, although all four paired runs show improvements and the average effect is positive, the difference does not reach statistical significance at the 0.05 level. Given the small number of runs and the variability in the gain magnitude, these results should be interpreted as evidence of a modest positive trend rather than a conclusive statistically significant improvement.

### 4.6. Visualization Analysis

Figure 5 presents qualitative visualization results of GCGFormer on representative remote sensing scenes containing dense, arbitrarily oriented, and large-aspect-ratio objects. The green boxes denote ground-truth annotations, the red boxes represent the baseline detector, and the blue boxes correspond to the predictions generated by the detector equipped with GCGFormer. As illustrated in Figure 5a–f, GCGFormer consistently produces more accurate localization and orientation estimation under complex geometric layouts.

Specifically, in densely distributed sports-field scenes shown in Figure 5c–e, the baseline detector exhibits noticeable localization deviations and orientation inconsistency, particularly for elongated courts with similar semantic appearance. In contrast, GCGFormer generates bounding boxes that align more precisely with object boundaries and preserve consistent directional structures. This improvement demonstrates that the proposed geometry-aware attention mechanism effectively captures orientation alignment and spatial structural relationships among neighboring objects. Figure 5g–i cover crowded ships, low-contrast maritime targets, and densely arranged aircraft with varying orientations.

In addition, Figure 5f shows that GCGFormer achieves superior discrimination capability in crowded vehicle scenes. The baseline detector tends to produce overlapping predictions and inaccurate angle estimation under dense arrangements, whereas GCGFormer maintains clearer object separation and more stable geometric localization. Similar improvements can also be observed in urban and playground scenes in Figure 5a,b, where the proposed method better suppresses background interference and preserves geometric consistency across different object categories and scales. Nevertheless, errors remain in scenes containing densely repeated objects with highly similar geometric attributes. In particular, adjacent ships, vehicles, or aircraft may receive high pairwise compatibility, resulting in excessive feature aggregation, inaccurate localization, or missed instances. These cases demonstrate that GCGFormer improves geometric consistency in many challenging scenes but cannot completely resolve ambiguities caused by low resolution, severe overlap, background interference, or highly repetitive spatial structures.

## 5. Discussion

The experiments show that GCGFormer provides modest overall improvements across the five evaluated detectors, with mAP gains ranging from 0.2 to 0.5 percentage points on the DOTA-v1.0 validation split. These results suggest that geometric compatibility can complement semantic attention under the evaluated settings. However, the class-wise effects are uneven. HC improves consistently across all detectors, whereas BR, LV, BC, and SBF improve in four of the five detectors. In contrast, ST decreases for four detectors, and relatively large degradation is observed for SH in some cases. This indicates that geometric priors may be less reliable for categories with large intra-class geometric variation or complex spatial arrangements.

The auxiliary geometric loss explicitly supervises the predicted orientation and aspect-ratio attributes, reducing their ambiguity and encouraging geometrically meaningful representations. Nevertheless, the reliability of these attributes has not been independently quantified using orientation or aspect-ratio prediction errors. Because other representative baselines with published results of these methods (such as LSKNet, ReDet, O^2^-DETR, and PP-YOLOE-R) commonly use the DOTA test set or different training strategies, they are not directly comparable with our locally reproduced validation-set results. Therefore, whether the gains of 0.2–0.5 mAP transfer to stronger baselines remains an open question.

GCGFormer introduces only 0.29 M additional parameters and 7.49 GFLOPs when integrated into Oriented R-CNN. However, constructing the N×N compatibility matrix has quadratic complexity with respect to the number of tokens. Future work will investigate more efficient geometric relation modeling, independently evaluate the reliability of the predicted attributes, and validate the method on stronger detectors and additional oriented-object datasets.

## 6. Conclusions

This study demonstrates that geometric information can guide self-attention effectively when expressed as explicit pairwise compatibility and adaptively balanced with semantic similarity. GCGFormer implements this insight at the feature-token level, allowing integration into two-stage, one-stage rotated detectors without redesigning their prediction heads. Matched experiments and four-seed evaluation on DOTA-v1.0 show modest but consistent improvements, with orientation alignment and the learnable Gate making important contributions. This design is practically relevant to remote-sensing scenes containing arbitrarily oriented and densely distributed objects while introducing only a small parameter increase. However, performance gains are not uniform across categories, geometric estimates remain imperfect, and the quadratic compatibility calculation reduces inference speed. Evaluation is also limited to one dataset. Future research will investigate sparse compatibility computation, uncertainty-aware geometric encoding, cross-dataset validation, and adaptation to other geometry-sensitive vision tasks.

## Figures and Tables

**Figure 1:**
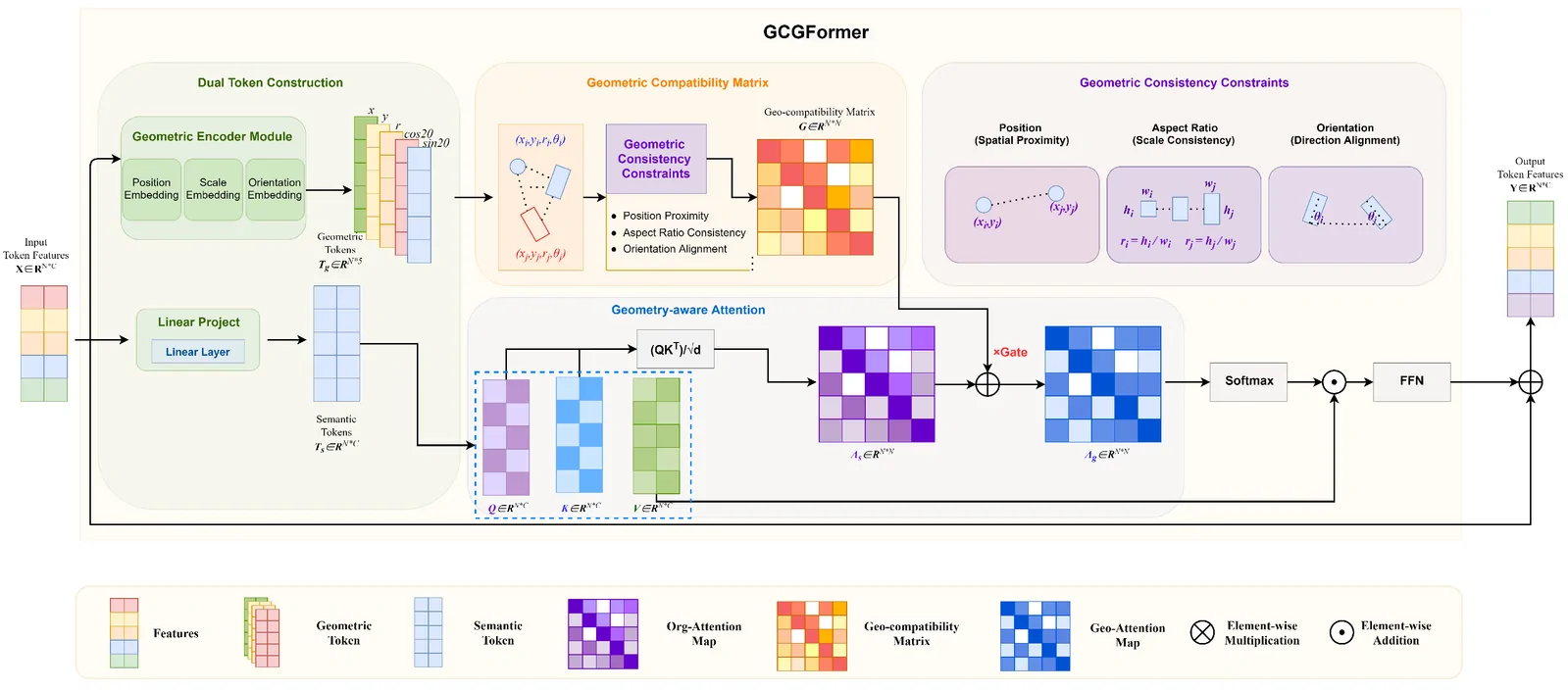
Overall architecture of the proposed GCGFormer module.

**Figure 2:**
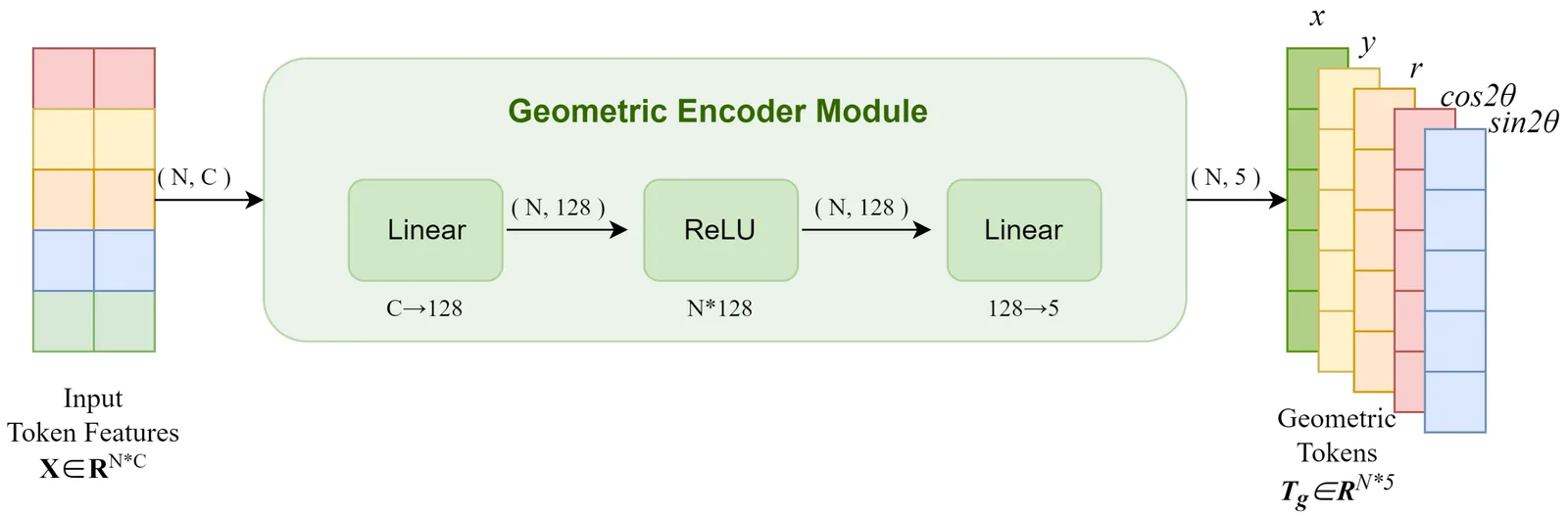
Architecture of the proposed GCGFormer Geometric Encoder.

**Figure 3:**
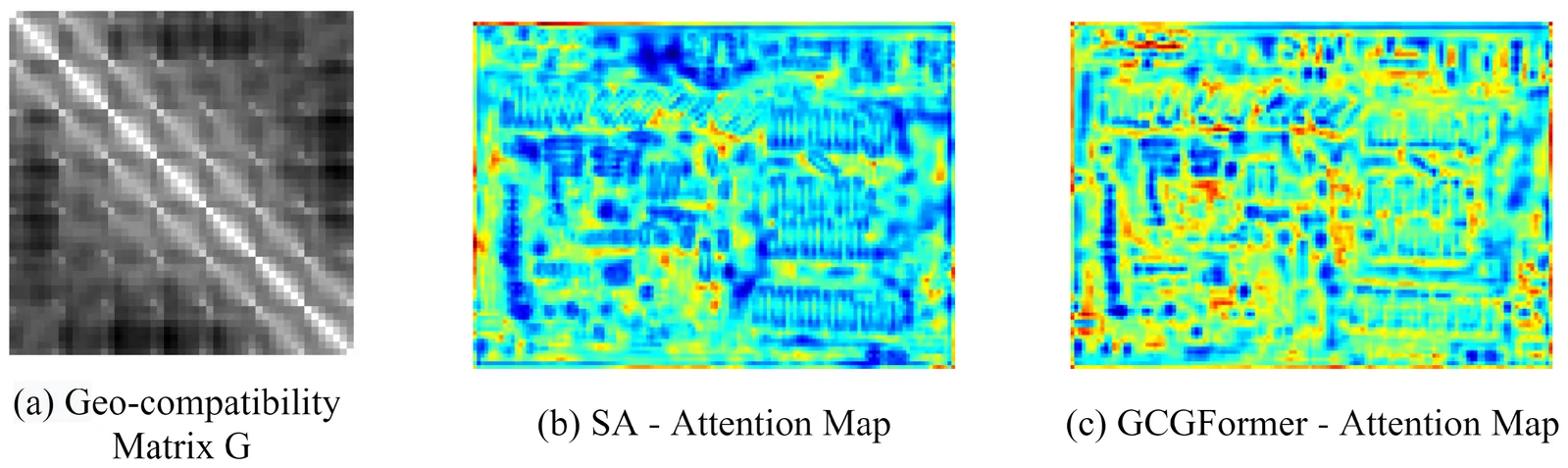
Visualization of geometric compatibility and attention responses using Oriented R-CNN on a representative DOTA-v1.0 validation image. (**a**) Fused geometric compatibility matrix *G*; (**b**) baseline self-attention map; and (**c**) geometry-guided attention map produced by GCGFormer.

**Figure 4:**
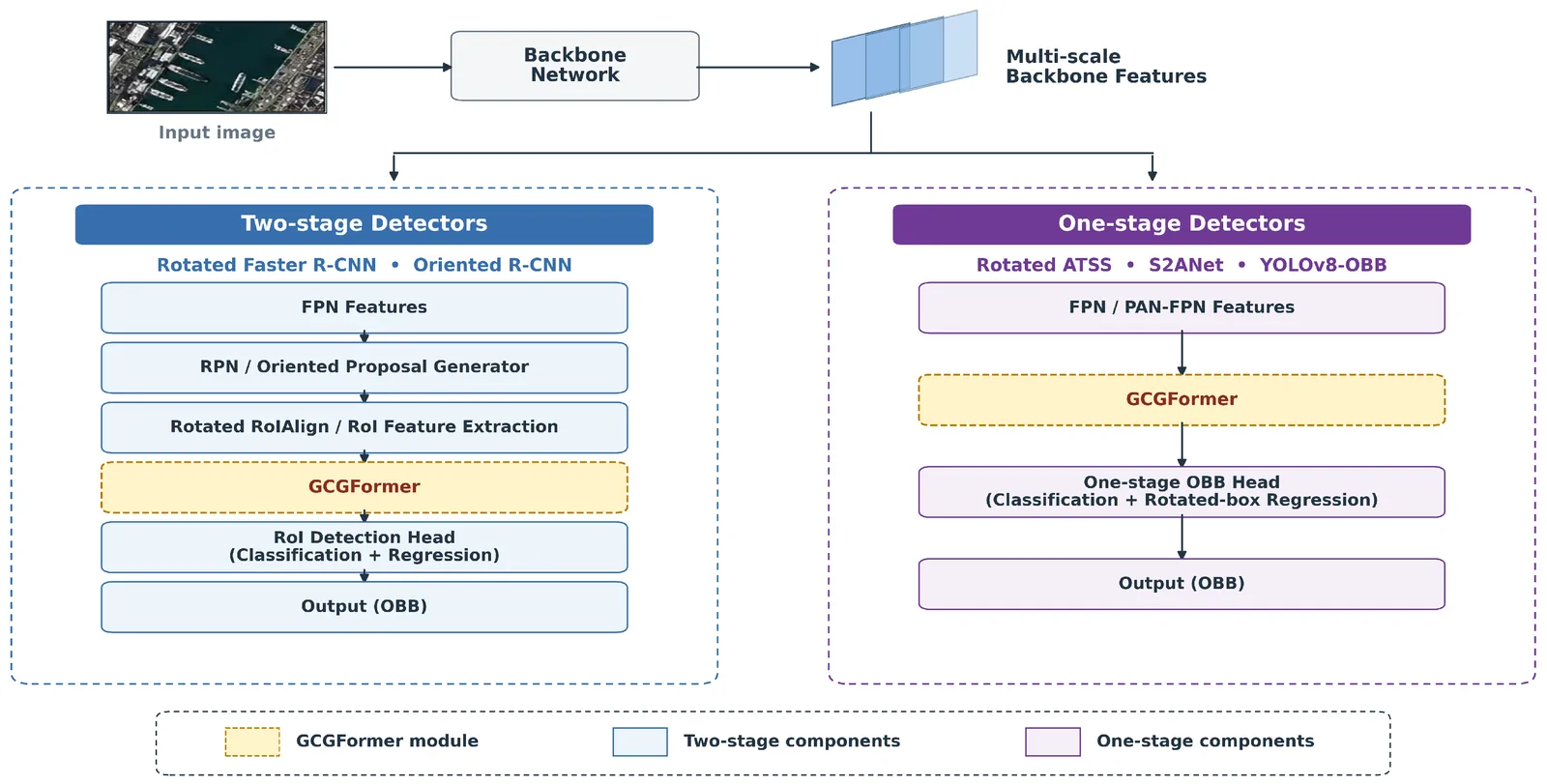
Integration of GCGFormer into two-stage and one-stage rotated object detectors. For Rotated Faster R-CNN-OBB and Oriented R-CNN, GCGFormer is inserted after Rotated RoIAlign and before the RoI detection head. For Rotated ATSS-OBB, S2ANet, and YOLOv8-OBB, it is placed after the FPN/PAN-FPN features and before the one-stage OBB detection head.

**Figure 5:**
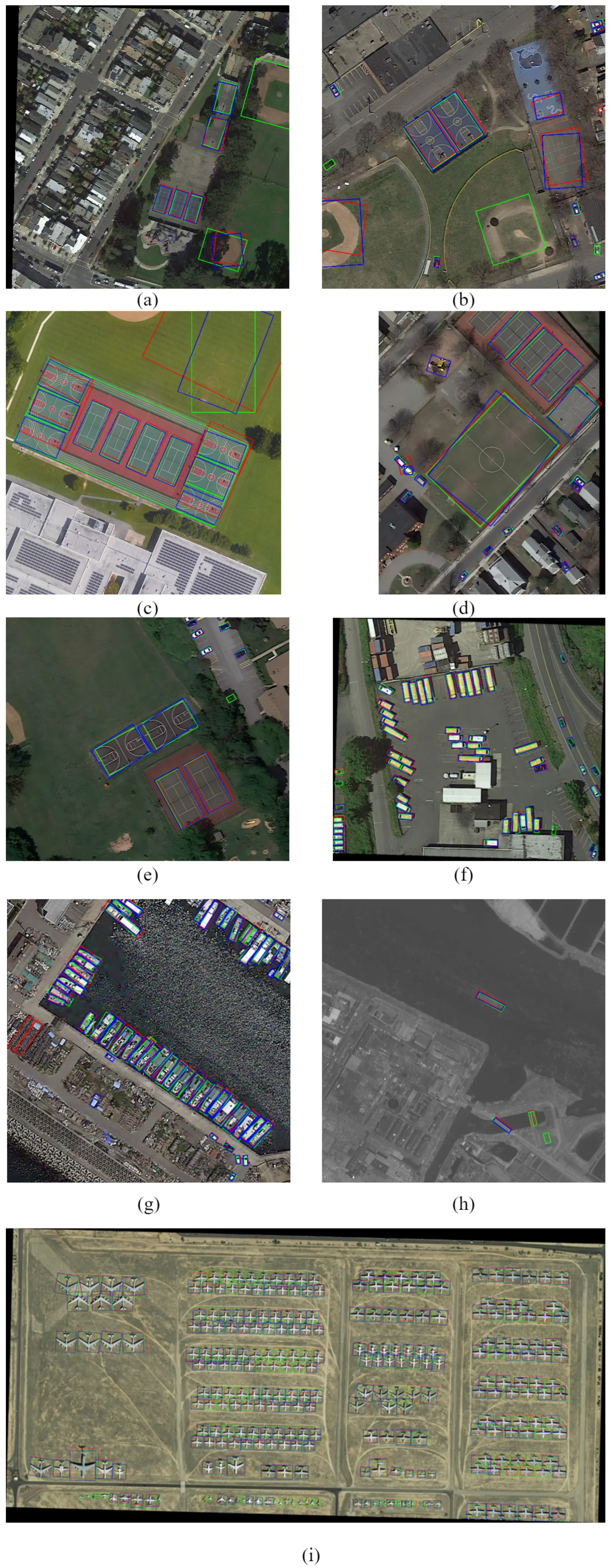
Qualitative visualization results of GCGFormer using Oriented R-CNN on representative remote sensing scenes on DOTA-v1.0 dataset (The green boxes denote ground-truth annotations, the red boxes represent the baseline detector, and the blue boxes correspond to the predictions generated by the detector equipped with GCGFormer).

**Table 1:** Ablation study and computational complexity of GCGFormer core component. (The baseline is Oriented R-CNN on the DOTA dataset (using Seed 4)).

Model	SA	GCG Former	mAP (%)	Gain	Params (M)	FPS	GFLOPS	Peak GPU (GB)
Baseline			65.1	–	41.95	32.13	199.58	2.7
Baseline	✓		65.2	+0.1	42.21	29.86	208.09	3.0
Baseline		✓	**65.6**	**+0.5**	42.24	26.64	207.07	3.1

The bold values represent the best performance.

**Table 2:** Ablation study of single factor. (The baseline is Oriented R-CNN on the DOTA dataset (using Seed 4)).

Model	Position	Aspect Ratio	Orientation	mAP (%)	Gain
Baseline				65.1	–
Baseline	✓			65.2	+0.1
Baseline		✓		65.1	+0.0
Baseline			✓	**65.5**	**+0.4**

The bold values represent the best performance.

**Table 4:** Ablation study of geometric weights and the gate mechanism in GCGFormer with Oriented R-CNN on the DOTA-v1.0 validation split.

Geometric Weights	Gate Mechanism	Seed	mAP (%)
Fixed equal weights 1/3 each	Learnable	4	65.3
Normalized learnable weights	Learnable	4	**65.6**
Normalized learnable weights	Fixed g = 1	4	65.2
Normalized learnable weights	Fixed g = 0	4	65.2

The bold values represent the best performance.

**Table 5:** Cross-detector comparison of published DOTA-v1.0 test-server results and locally evaluated results on the DOTA-v1.0 validation split (using Seed 4).

Detector	Optimizer	Published	Our Reproduced	Reproduced + GCGFormer	Gain
Rotated Faster R-CNN [33,34]	SGD	73.4	63.3	63.8	+0.5
Oriented R-CNN [35]	SGD	75.7	65.1	65.6	+0.5
Rotated ATSS [36]	SGD	70.6	61.7	62.1	+0.4
YOLOv8-OBB [37]	AdamW	78.0	72.7	72.9	+0.2
S2ANet [38]	SGD	73.9	62.7	63.1	+0.4

**Table 6:** Category-wise AP and overall mAP comparisons between the in-house baselines and their corresponding GCGFormer-enhanced models on the DOTA-v1.0 validation split (using Seed 4).

Detector	mAP	PL	BD	BR	GTF	SV	LV	SH	TC	BC	ST	SBF	RA	HA	SP	HC
Rotated Faster R-CNN [33,34]	63.3	80.6	74.8	41.7	63.1	58.0	74.2	78.4	90.1	54.7	53.6	53.7	51.2	66.3	55.1	54.0
+GCGFormer	63.8	80.7	73.2	41.0	64.8	57.6	75.4	71.2	89.7	61.7	53.4	58.0	52.1	66.8	54.9	56.6
Oriented R-CNN [35]	65.1	80.8	75.3	44.0	69.2	59.3	77.8	80.0	90.1	61.5	53.6	59.9	49.7	67.7	53.3	54.4
+GCGFormer	65.6	80.7	73.9	44.3	68.2	58.9	78.1	80.1	90.2	62.0	53.4	60.8	52.3	67.9	53.9	59.2
Rotated ATSS [36]	61.7	80.5	68.7	41.6	65.7	56.3	74.6	75.0	89.5	54.0	59.9	49.2	50.3	61.1	51.8	46.7
+GCGFormer	62.1	80.5	69.0	42.2	63.8	55.0	74.5	75.4	89.4	58.6	60.0	49.5	49.6	60.7	52.6	50.6
YOLOv8-OBB [37]	72.7	87.5	84.2	67.2	66.5	66.5	81.0	82.2	90.2	66.9	81.1	47.2	73.9	81.1	54.6	59.8
+GCGFormer	72.9	86.2	83.1	67.9	63.9	70.7	81.6	81.8	91.1	66.7	80.3	46.2	73.0	81.3	51.9	67.6
S2ANet [38]	62.7	80.7	68.2	41.8	60.2	57.7	75.9	78.7	89.2	56.7	61.2	50.7	52.3	64.6	52.2	50.3
+GCGFormer	63.1	80.6	67.9	42.6	60.9	57.5	76.1	78.9	90.0	58.0	60.7	52.3	51.0	64.5	50.9	54.1

**Table 7:** Comparison with representative published methods on the DOTA-v1.0 test set. The results are quoted from the corresponding publications and are provided only to indicate the performance range of recent methods. They are not directly comparable with our locally evaluated validation-set results (using Seed 4).

Method	Reported Setting	Evaluation Split	mAP (%)
ReDet [29]	Multi-scale testing	Test	80.10
O^2^-DETR [32]	Cascaded fine-tuning	Test	79.66
PP-YOLOE-R-x [31]	Multi-scale training and testing	Test	80.73
LSKNet [30]	Best reported configuration	Test	81.85

**Table 8:** Statistical robustness of the reproduced baseline and GCGFormer-enhanced models on the DOTA-v1.0 validation split over four random seeds. (The baseline is Oriented R-CNN on the DOTA dataset (using Seed 4)).

Model	Seed42	Seed2006	Seed13	Seed4	Mean ± SD
Baseline	65.06	64.69	65.11	65.10	64.99 ± 0.20
+GCGFormer	65.16	65.49	65.24	65.60	65.38 ± 0.21
Gain	+0.1	+0.8	+0.13	+0.50	+0.39 ± 0.33

## Data Availability

The source code is publicly available at https://github.com/Begonia060410/GCGformer (accessed on 14 September 2026). The DOTA datasets can be obtained from their official provider subject to the corresponding license.

## Appendix A

**Algorithm A1** GCGFormer: Geometry-Compatibility-Guided Attention
**Require:** Feature tokens $X\in R^{B\times N\times C}$; matched ground-truth OBBs during training **Ensure:** Geometry-enhanced features *Y*; auxiliary geometric loss $L_{\mathrm{geo}}$ during training 1: Generate semantic tokens: $T_{s}\leftarrow \phi_{s}\left(X\right)$ 2: Generate geometric tokens: $T_{g}\leftarrow \phi_{g}\left(X\right)$ 3: Obtain normalized spatial positions $s_{i}$, orientation vectors $o_{i}$, and aspect ratios $r_{i}$ from $T_{g}$ 4: **if** training **then** 5: Assign each positive region to its matched ground-truth OBB 6: Construct the ground-truth orientation vector $o^{*}\leftarrow \left[\cos\left(2\theta^{*}\right),\sin\left(2\theta^{*}\right)\right]$ 7: Obtain the ground-truth aspect ratio $r^{*}\leftarrow W^{*}/H^{*}$ 8: Compute Gaussian spatial weights $s^{*}$ 9: Compute the auxiliary geometric loss $L_{\mathrm{geo}}$ 10: **else** 11: $L_{\mathrm{geo}}\leftarrow 0$ 12: **end if** 13: Compute query, key, and value: $Q,K,V\leftarrow \phi_{q}\left(T_{s}\right),\phi_{k}\left(T_{s}\right),\phi_{v}\left(T_{s}\right)$ 14: **for** each token pair (i,j) **do** 15: Compute position compatibility $G_{p}\left(i,j\right)$ 16: Compute orientation compatibility $G_{o}\left(i,j\right)$ 17: Compute aspect-ratio compatibility $G_{r}\left(i,j\right)$ 18: **end for** 19: Normalize the geometric weights: $$\left[w_{p},w_{o},w_{r}\right]\leftarrow \mathrm{Softmax}\left(\left[a_{p},a_{o},a_{r}\right]\right)$$ 20: Construct the geometric compatibility matrix: $$G_{ij}\leftarrow w_{p}G_{p}\left(i,j\right)+w_{o}G_{o}\left(i,j\right)+w_{r}G_{r}\left(i,j\right)$$ 21: Compute semantic-attention logits: $$S\leftarrow \frac{QK^{⊤}}{\sqrt{d}}$$ 22: Compute normalize geometry-aware attention: $$A_{g}\leftarrow \mathrm{Softmax}\left(A_{s}⊕\left(\mathrm{Gate}\cdot G\right)\right)$$ 23: Generate geometry-enhanced features: $$Y=A_{g}V$$ 24: Apply the residual connection and FFN: $$Z\leftarrow T_{s}+Y+\mathrm{FFN}\left(T_{s}+Y\right)$$ 25: **return** $Z,L_{\mathrm{geo}}$
